# Brain Abscess Arising From Pansinusitis in an Immunocompetent Young Adult: A Diagnostic Challenge

**DOI:** 10.7759/cureus.103155

**Published:** 2026-02-07

**Authors:** Stanley W Sun, Matthew Gordon-Short, Chinmay Patel, Eric H Chou

**Affiliations:** 1 Department of Emergency Medicine, Baylor Scott and White All Saints Medical Center, Fort Worth, USA; 2 Department of Emergency Medicine, Burnett School of Medicine at Texas Christian University, Fort Worth, USA; 3 Department of Emergency Medicine, Taipei Hospital, Ministry of Health and Welfare, New Taipei City, TWN

**Keywords:** acute sinusitis, frontal mucocele, methicillin-sensitive staphylococcus aureus, polymicrobial brain abscess, refractory headache, streptococcus anginosus group

## Abstract

Brain abscesses are rare but potentially life-threatening infections that require prompt recognition and treatment. Although they typically occur in patients with identifiable risk factors, they can occasionally present in otherwise healthy individuals. Sinusitis is a recognized source of intracranial infection, but its progression to a brain abscess in immunocompetent patients is uncommon and may be clinically subtle. We report the case of a 21-year-old previously healthy male who presented to the emergency department (ED) with a non-specific bifrontal headache. He was initially diagnosed with migraine and discharged. Eight days later, he returned with worsening symptoms, including photophobia and fatigue. Imaging revealed a mucocele involving multiple paranasal sinuses and a right frontal lobe brain abscess with associated meningitis.

Cultures from surgical drainage grew *Streptococcus anginosus* and *Staphylococcus aureus*. The patient underwent surgical abscess drainage and sinus surgery, followed by intravenous antibiotics, with complete recovery. This case highlights the importance of maintaining a high index of suspicion for intracranial complications of sinusitis, even in immunocompetent patients with non-specific symptoms. Timely neuroimaging and multidisciplinary management are essential for favorable outcomes in such presentations.

## Introduction

Brain abscesses are the most common form of focal suppurative intracranial pathology, although brain tumors remain approximately fifty times more prevalent [1]. Despite their rarity, brain abscesses account for about 1 in 10,000 hospital admissions [1,2]. A brain abscess is defined as a localized collection of pus within the brain parenchyma, resulting from either contiguous spread of infection or hematogenous dissemination from distant sources [3,4]. Chronic sinusitis and mucoceles are among the common local sources [5]. If left untreated, mucoceles may present with a range of rhinologic and neurologic symptoms such as headache, nasal discharge, dizziness, seizures, visual changes, or signs of frontal lobe dysfunction [5,6]. In rare cases, mucoceles may erode intracranially and facilitate infectious spread [6].

The most frequently isolated organisms in brain abscesses include *Staphylococcus aureus* and members of the Viridans group streptococci [4,7]. Among the latter, the *Streptococcus anginosus* group (SAG), including *S. anginosus, S. intermedius*, and *S. constellatus, *is particularly known for its abscess-forming tendency in various body sites [8,9].

The clinical presentation of intracranial pathology is highly variable, as are the associated prognoses and treatment strategies. Emergency physicians must exercise a high degree of vigilance when evaluating patients with potential intracranial involvement, as timely and accurate diagnosis is critical. Historically, brain abscesses carried a mortality rate as high as 60% prior to the 1970s [10-12]. However, advances in antimicrobial therapy and neuroimaging have significantly improved outcomes, with recent case series reporting mortality rates between 8% and 25% [10,13-15]. This report presents a case of a young, immunocompetent patient diagnosed with a brain abscess due to *Streptococcus anginosus, *believed to have originated from the contiguous spread of pansinusitis. This report highlights the clinical features, diagnostic process, and multidisciplinary management of a *Streptococcus anginosus *brain abscess in a previously healthy young adult.

## Case presentation

History

A 21-year-old male with a history of asthma presented to the emergency department (ED) with a severe bifrontal headache for 2 days. Neurologic examination at that time was nonfocal, and his symptoms improved with multimodal therapy, including acetaminophen, ketorolac, and methocarbamol. He was discharged with a diagnosis of migraine and instructed to follow up with his primary care provider for further evaluation. Eight days later, he returned to the ED with a persistent bifrontal headache associated with photophobia and fatigue, reporting progressive worsening since the initial visit. He denied fever, chills, myalgias, chest pain, shortness of breath, nausea, vomiting, or diarrhoea. He also denied focal neurologic symptoms, including facial droop, slurred speech, unilateral weakness, dizziness, vertigo, or gait disturbance.

Physical examination

On presentation, vital signs were notable for a heart rate of 96 beats per minute and blood pressure of 116/75 mmHg. The patient was afebrile. Physical and neurologic examinations were unremarkable, with no meningeal signs or focal neurologic deficits.

Diagnostic assessment

**Figure 1:**
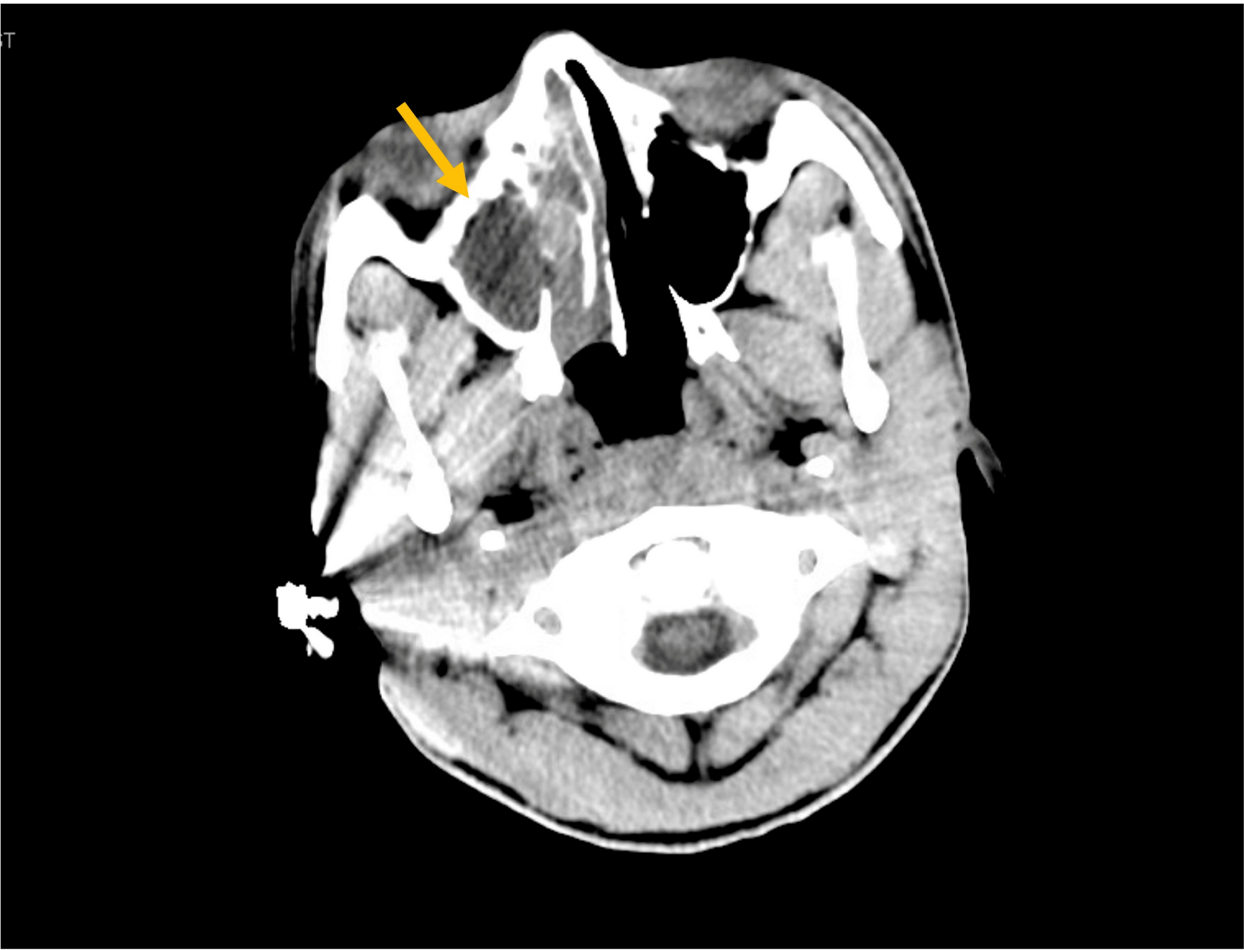
Non-contrast CT scan showed a large degree of opacification with what appears to be expansion suggestive of a mucocele involving the right frontal, right ethmoid and right maxillary sinuses.

Laboratory evaluation was significant for leukocytosis (white blood cell count 17.2 × 10³/μL; reference range 4.5-11.0 × 10³/μL) with neutrophil predominance (absolute neutrophil count 14.51 × 10³/μL, 81.1%). All other laboratory values were within normal limits. Computed tomography (CT) of the sinuses demonstrated a large mucocele involving the right frontal, ethmoid, and maxillary sinuses (Figure 1).

**Figure 2:**
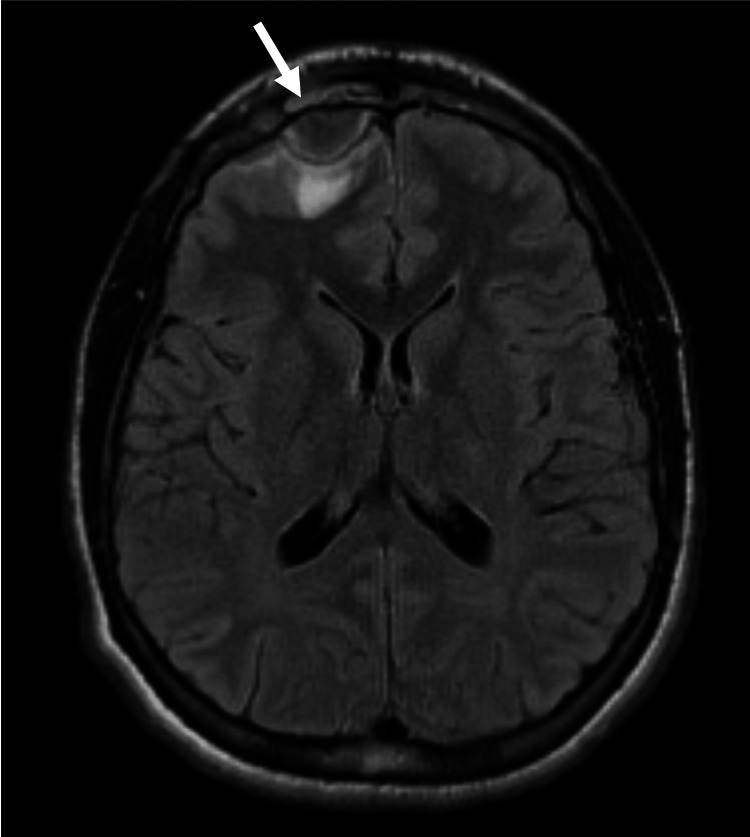
MRI showed 2 cm intracranial abscess at the right frontal convexity with mass effect on the adjacent right frontal lobe and adjacent cerebral edema and potentially cerebritis.

Otolaryngology was consulted, and intravenous ampicillin-sulbactam was initiated. The patient was admitted for further evaluation. Subsequent magnetic resonance imaging (MRI) of the brain revealed a right frontal lobe abscess with associated meningitis (Figure 2).

Therapeutic intervention

Neurosurgery was consulted, and the patient underwent a bicoronal craniotomy with surgical drainage of the abscess, in conjunction with endoscopic sinus surgery, including ethmoidectomy.

Intraoperative cultures yielded *Streptococcus anginosus *and* Staphylococcus aureus*. Based on recommendations from the infectious diseases service, antimicrobial therapy was narrowed and transitioned to intravenous ceftriaxone, vancomycin, and metronidazole.

Follow-up and outcomes

The patient was discharged after placement of a peripherally inserted central catheter (PICC) to facilitate completion of a planned 28-day course of intravenous antimicrobial therapy with ceftriaxone, vancomycin, and metronidazole. At outpatient follow-up following completion of the planned 28-day antimicrobial course, the patient demonstrated sustained clinical improvement with complete resolution of headache and no focal neurological deficits. He remained asymptomatic, with no recurrent headache, neurological deficits, or infectious symptoms.

## Discussion

Headache is one of the most common reasons for ED visits and is most frequently attributed to benign primary headache disorders, particularly in young, otherwise healthy patients [7,16-18]. In the absence of focal neurological deficits, fever, or meningeal signs, clinicians may reasonably defer advanced neuroimaging. However, this case underscores the importance of maintaining vigilance when headaches are persistent, progressive, or atypical. Furthermore, seemingly benign headache presentations warrant close outpatient follow-up and consideration of neuroimaging. Our patient initially presented with a severe bifrontal headache and a non-focal neurological examination, leading to a diagnosis of migraine and discharge. His subsequent return with persistent and worsening symptoms highlights a well-recognized diagnostic challenge: early intracranial infections may present subtly and evolve over days. Systemic abnormalities, such as leukocytosis in this case, can serve as early warning signs and should prompt reconsideration of the diagnosis and further evaluation. Prior studies emphasize that repeat presentations for the same complaint are independently associated with higher rates of serious underlying pathology and warrant reassessment rather than diagnostic anchoring [12,18-20].

Frontal sinusitis-associated brain abscesses are uncommon but well-described in the literature and occur across a wide age range (Table 1). Clinical presentations are highly variable, ranging from mild headache to seizures, focal neurological deficits, or altered mental status. Importantly, several reported cases, including adolescents and young adults, initially presented with isolated headache or nonspecific symptoms, similar to our patient’s early course [17-19]. These findings reinforce that the absence of dramatic neurological findings does not exclude significant intracranial disease. Notably, two adolescent patients (ages 14 and 16) were evaluated primarily for persistent headache with low-grade systemic symptoms, mirroring the subtle early course observed in our patient [1,18,19].

**Table 1:** Summary of reported cases of intracranial abscess associated with paranasal sinusitis. The table includes patient demographics, initial clinical presentation, treatment modalities, including antimicrobial therapy, surgical interventions, and postoperative management, and documented outcomes based on follow-up imaging or clinical status. M: male; F: female; MRI: magnetic resonance imaging; CT: computed tomography; GCS: Glasgow Coma Scale.

	Year	Country	Age/Gender	Initial Presentation	Treatment	Outcomes
Iacono, et al. [17]	2020	Italy	16 M	Headache, right upper eyelid edema, photophobia	Ceftiaxone, amphotericin B, rifampicin, -> Craniotomy -> Ceftriaxone, amphotericin B, rifampicin, Vancomycin, dexamethasone,	5-month MRI: resolution of abscess
Yang, et al. [18]	2024	China	14 M	Headache, fever, rhinorrhea, dizziness	Ceftriaxone, Vancomycin -> open drainage of frontal and maxillary sinuses -> Vancomycin and meropenem	3-month MRI: resolution of abscess
Michali et al. [19]	2021	Greece	39 M	Headache, fever, rhinorrhea	Ceftriaxone, clindamycin, acyclovir, antiepileptics -> craniotomy	6-month MRI: resolution of abscess
Weidmayer [5]	2015	USA	68 M	Intermittent diplopia, chronic sinusitis	Endoscopic right ethmoidectomy, frontal sinusotomy	2-month follow-up appointment: asymptomatic
Tani [20]	2024	China	43 M	Fever, vomiting	Ceftriaxone -> Brain stereotactic abscess puncture & drainage -> Ceftriaxone, vancomycin	Asymptomatic at future follow-up
		China	63 F	Dizziness	Emergent brain stereotactic abscess puncture & drainage -> Ceftriaxone	Post-op day 34 follow-up: asymptomatic
		China	58 M	Incidental finding	Brain stereotactic abscess puncture & drainage -> Ceftriaxone	2-month CT: partial absorption of gas and edema
Naik et al. [21]	2023	USA	67 M	Forehead mass, facial pain, nasal drainage	Ampicillin-sulbactam -> Bi-frontal craniotomy with abscess drainage and pericranial flap -> Ceftriaxone, metronidazole	Lost to follow-up
Esplin et al. [4]	2017	USA	70 M	Found by EMS GCS 8, facial droop, started having seizures	Emergent craniotomy -> IV antibiotics	Discharged post-op day 8 with improvement in neurological symptoms
Niehaus et al. [22]	2018	USA	49 M	Multiple seizures requiring intubation	Craniotomy -> Ceftriaxone, metronidazole, vancomycin	Mild left hemiparesis and hemiataxia, discharged on hospital day 13 to acute rehabilitation

Imaging plays a central role in diagnosis. While a CT scan is often the first-line modality in the ED, magnetic resonance imaging provides superior sensitivity for detecting intracranial complications, including abscess formation and meningitis [19,20]. In this case, sinus CT identified an extensive mucocele, prompting speciality consultation and admission, while subsequent MRI revealed the full extent of intracranial involvement. This sequential approach reflects real-world practice and highlights the importance of escalating imaging based on evolving clinical context.

Management of sinusitis-related brain abscesses almost universally requires a combined medical and surgical approach [4,5,19,21,22]. In reported cases, early neurosurgical drainage, via craniotomy or stereotactic aspiration, combined with endoscopic sinus surgery and prolonged broad-spectrum antimicrobial therapy, has been associated with favorable outcomes. Our patient underwent prompt surgical intervention and received targeted antimicrobial therapy guided by culture results, leading to complete clinical recovery without neurological sequelae.

The isolation of *Streptococcus anginosus* further supports the pathogenic link between sinonasal disease and intracranial abscess formation [9,21]. Although a commensal organism of the oral and gastrointestinal flora, *S. anginosus* is well known for its propensity to cause invasive pyogenic infections, particularly abscesses of the brain, liver, and lungs. Its presence in this case aligns with prior reports and reinforces the importance of empiric coverage for anaerobic and streptococcal species in suspected intracranial infections of sinus origin [21,22]. Ultimately, this case illustrates how a common complaint can mask a life-threatening condition. It highlights the dangers of diagnostic anchoring, the value of reassessment in patients with persistent symptoms, and the importance of integrating clinical gestalt with objective data. A multidisciplinary approach involving emergency medicine, otolaryngology, neurosurgery, and infectious diseases was critical to achieving a favorable outcome. Clinicians should maintain a low threshold for repeat evaluation and advanced imaging when headaches deviate from their expected course, even in immunocompetent patients without classic red-flag features.

## Conclusions

 This case underscores the need for a high index of suspicion for intracranial complications of sinusitis, even in immunocompetent patients with nonspecific symptoms. Prompt neuroimaging and multidisciplinary care are critical for favorable outcomes. Early recognition and timely surgical and antimicrobial management can prevent neurological deterioration and long-term sequelae. Emergency physicians should maintain awareness of this rare but serious complication when evaluating patients with persistent headache, fever, or focal neurological findings in the setting of sinus disease.
